# The Pluriverse of Intoxication: Words, Lives, Worlds in Islamicate History

**DOI:** 10.1086/721659

**Published:** 2022-09-01

**Authors:** Maziyar Ghiabi

**Affiliations:** Maziyar Ghiabi is senior lecturer in medical humanities and politics at the University of Exeter and director of the Center for Persian and Iranian Studies at the Institute of Arab and Islamic Studies, Exeter

## Abstract

This article establishes a conceptual framework for decolonial drug histories and, at the same time, moves beyond decolonization. It brings back the radical alterity of historical figures of intoxication in the Islamicate world and introduces them as paradigms with potential to go beyond decolonization. This approach refers to the urgency of not comfortably relying on decolonial critique as moral indignation toward the past but rather showing that drug histories subsume radically different epistemologies and ontologies from those enunciated by coloniality/modernity. The article studies the Islamicate world through a decolonized nomenclature based on everyday historical approaches, beyond the myths of quintessential intoxicated Orient or as the inherent space of religious prohibitions. By introducing alternative epistemologies on mind-altering substances and their radical ontologies, this experiment in writing history as world-building shows how non-Western knowledge and practice can make other realities—and histories—possible.

To cure your ignorance / drop opium inyour wine.

Rumi, *Divan-e Shams*^1^

One of the priests had explained to the natives (naturally the account refers to them as sauvages) that the high mortality rate they were suffering was due to wine and spirits which they didn’t know how to use in moderation. “Why don’t you write to your great King, “ one of the natives said, “that he prohibit these beverages from being brought in, since they kill us?” The Jesuit replied that the French needed these drinks in order to face the oceanic voyages and incredible cold of these regions. “Then make it such, “ the other replied “that only they drink it.” At this point, another native stood up: “No, it’s not these beverages which take life away from us, it is your writings: since you first described our country, our rivers, our lands, our forests, all of us began to die, as did not occur before your arrival.”

French Jesuit Paul de Brebeuf’s letter to the provincial priest of the company regarding events in Québec 1636^2^

Can we think histories of drugs without the idea of the West? And, after all, should we do so?

This is a question that needs addressing when attempting to write decolonial drug histories. Our ideas about “drugs” and their intimate biomedical compound, “addiction” or “intoxication,” are intrinsically linked to the making of the idea of the West. Both concepts emerged in the late eighteenth century and fully developed in the nineteenth century amid the colonial onslaught and the institutionalization of modernity and its capitalist forms of life. Since then, the West and drugs have co-constituted ideas and narratives about the “non-West.” They have made possible mythologizing about the colonial world, the “Global South,” and, ultimately, the “Other.”

Now, as Boaventura de Sousa Santos puts it, we have realized that “the understanding of the world far exceeds the Western understanding of the world; [that] there is no global justice without global cognitive justice [and that] the emancipatory transformations in the world may follow grammars and scripts other than those developed by Westerncentric critical theory.”^3^ In embracing the invitation to think with the epistemologies of the South, I do not seek to formulate new theories in this article; rather, I experiment with a different form of theorizing, in dialogue with a geocultural environment that has been abandoned or ignored when pursuing critical thinking—namely, the Islamicate world.^4^

Histories and mythologies become entangled when drugs are seen in the Islamicate world through an epistemic forcing onto an everyday lifeworld.^5^ Historian Joseph A. Massad argues that Islam is one of the conditions for the emergence of the idea of the West—of Europe, more precisely—and of the birth of liberalism. It is “an internal constituent of liberalism,” he says, echoing Edward Said’s intuition that “the Orient is an integral part of European material civilisation and culture.”^6^ In parallel, drug scholar Toby Seddon argues that drugs and freedom are essential formative notions of modernity and politics.^7^ It turns out that the ways Western, colonial knowledge producers understood Islam and drugs have enabled and forged the way they have made sense of their own selves and of the West; therefore, narratives about drugs and Islam are foundational to how the West imagines itself rather than to how the Other or drugs are imagined.

“Drugs” as an epistemic category belongs to the modern world—to what Walter Mignolo popularized as coloniality/modernity, the matrix of power/knowledge that emerged from the sixteenth century.^8^ As a colonial category, “drugs” could not capture the multitude of mind-altering substances and the experiences of intoxication existing outside the West. It could not represent the pluriversal ontologies of the non-West. The term “drug” became the prelude to the international drug control system that emerged in the twentieth century and that progressively captured the multitude of substances not conforming to Western cultures of consumption and capitalist exploitation. The origin of the term “drug,” however, did not lie in scientific pursuits or the imperial metropoles’ legal reforms; instead, it was the idea/myth of drugs originating in the Orient, in the colonies, that provided epistemic force for this political project. Through the association of drugs, and addiction, to the Other—in the guise of the Indian, African, Arab, Turk, Persian, or Muslim more generally—Western knowledge producers and their governments marked their ontological difference vis-à-vis those colonized, the “inferior races.”^9^

## I

The aim of this article is twofold: to provide a conceptual approach to writing decolonial drug histories and, at the same time, to attempt moving beyond decolonization itself. Its purpose is not rooted in empirical historicity, although it relies on the work of social and cultural historians of drugs and alcohol.^10^ Rather it introduces a series of epistemological considerations that could help writing drug histories through new ways of theorizing rather than through the application of theories or by uncovering unstudied histories through a Western or West-centric gaze. To do so, this article calls for adopting three epistemological steps:

1. Rethinking of the names and words we use in writing drug histories, making a case for an alternative (decolonial) philology. This initial epistemological step comes with the act of understanding why we use certain words rather than others, words being the wanting and yet still indispensable tools of the way we make knowledge today. It also considers forms of ignorance production and reification on drugs and how this has obscured localized epistemologies on intoxication.
2. Reclaiming the historical centrality of the everyday as a site and time to understand the histories of drugs and alcohol in the Islamicate (and colonial) world. The study of everyday history offers ways of entering the shaded, dim-lighted, dusty interstices of the life and lifeworlds of drugs, where grand historical forces such as colonialism and its allies (imperialism and capitalism) have not come to impose hegemonic uniformity and univocality. The everyday is where coloniality/modernity has not scrupulously overwritten the phenomenological script of non-Western peoples. In the everyday, non-Western peoples become historical agents rather than objects of colonial ignorance/knowledge making.
3. Unearthing a historical and epistemic figure of intoxication from thirteenth-century Iran, known as the *rend*, as bearing the potential to move beyond West-centric scripts. It is an experiment in writing (drug) history as a form of world building, showing how non-Western knowledge and practice made other realities—and histories—possible.

The article does not set a new universal vocabulary, and it does not provide a versatile conceptual toolkit to ease the plight of historical research. The works of feminist, new materialist, and science and technologies studies (STS) scholars who have produced a critique of Western rationalism and positivist reason provided ground for intellectual dialogue.^11^ Cultural studies scholars and anthropologists have respectively called for epistemological alterity and/or an ontological turn in engaging with the world, whereas drug scholars have spoken of “assemblages” (as formulated by Gilles Deleuze) to capture the contingency of drug “problems” through the multilayered action of language, policy, and health.^12^ In this vein, the article’s major contribution is to show the possibility of a decolonial drug history through the encounter of words (language), life (everyday), and new epistemic and ontological experiences in the Islamicate world—the latter having so far been the terra incognita of historical scholarship on drugs.^13^ In doing so, I do not dwell too long on critique of drug colonialism as such (this having been already explored in some detail by historians).^14^ Rather, my aim is to bring back the radical alterity of historical figures of intoxication in the Islamicate world and introduce them as paradigms having the potential to move drug history beyond decolonization.^15^ This latter step is what moves the article toward proposing how to think of a pluriverse of intoxication beyond existing (inter)disciplinary debates within decolonial, anthropological, and drug theory while engaging with lines of thought within these fields.

By going beyond decolonization, this article refers to the urgency to not comfortably fix on decolonial critique as moral indignation toward the past but rather to show that drug histories subsume radically different epistemologies and ontologies from those enunciated by coloniality/modernity. By introducing alternative epistemologies on mind-altering substances (i.e., drugs) and their radical ontologies, the article studies the Islamicate world beyond the myths of the quintessential intoxicated Orient or as the inherent space of religious prohibitions. Rather than contention with existing critical scholarship, this article tries to air and ground the possibilities of new conversations about intoxication and human experience by reclaiming the historical and ethical potentials of unorthodox lives in the Islamicate world. In turn, this approach attempts to take the study of intoxication and drugs beyond the question of objects, commodities, assemblages, or “lost and unheard voices” and to traverse into ambiguous territories where our understanding of intoxication involves ethics beyond morality and politics beyond society—that is, how living can shape the experience of the world.

This decolonial approach to the Islamicate world of intoxication should not be confused with an attempt to Islamize drug history or to see the latter through a religious lens. Frantz Fanon’s warning that decolonization should not be confused with straightforward indigenization or a rewriting of history as a celebration of the local remains a guiding principle in this article.^16^

### First step: Naming the “DRUG”

Attempts at decolonizing histories cannot avoid dealing with the most basilar tenets of an epistemic framework: words and names. Rather than digging out new historical truths, this archaeological approach “excavates the premises that qualify knowledge as truth in a given period and the ‘subjugated knowledges’ these premises disqualified.”^17^ This method is not concerned with the “subalterns per se” but with the “conscious desire to remain non-elite” and to explore (with) those “who did not possess, or did not want, the power to produce texts.”^18^

The (epistemic) force of coloniality/modernity is visible through the naming of things in the non-West. Massad argues that “one of the difficulties in analysing what Islam has come to mean and to refer to since the nineteenth century is the absence of agreement on what Islam actually is.” He then asks, “Does Islam name a religion, a geographical site, a communal identity; is it a concept, a technical term, a sign, a taxonomy?” He concludes that this conceptual bundle is compounded “by the fact that Islam has acquired referents and significations it did not formerly posses.”^19^ The name “drugs” has a chronology that intersects with that of Islam, both being the outcome of epistemic forcing on realities and forms of knowledge that previously encapsulated ordinary, medical-scientific, ethical-philosophical, and literary dimensions; and which by the nineteenth century were progressively reduced to emblems of the Other, antithesis of the liberal West.

This brings us to the politics of words. Categories that enable knowledge—epistemic pursuits, such as the writing of history—are always and necessarily (alas!) spoken in words, in language (it would be revolutionary to write history by dancing or using shades on a wall). Words and their use are never uninfluential in this sense. They are substantial by the mere fact of being (used). Giorgio Agamben may have referred to this when writing that “a name is a way of being [*modo di essere*] of the thing being named. The potential/power [*potenza*] of being thought in the name.”^20^ For our purpose, we need to ask what happens to the things of the world, such as drugs, “because of the existence of names.”^21^ It is not sufficient to qualify nouns and words with adjectives (“illicit,” “illegal,” “medical,” “recreational,” etc.). Nouns establish the horizon of that which is “sayable, credible, legitimate, or realistic” and vice versa, the things we believe in are given names.^22^ Adjectives do not allow us to discuss the terms of the discussion because they do not question the hegemonic force of words and nouns, such as “drugs.”

How did the word “drug,” then, come into use? Jacques Derrida referred to “drugs” as a moral and political construct closely knit with the purpose of prohibition.^23^ Toby Seddon claims that it is an invented concept that is symbiotic with the global drug prohibition system.^24^ To support his claim, he traces a genealogy of the term in the English language, recognizing that to ascertain a stronger, comprehensive claim, it would be necessary to discuss genealogies other than that of the English language. Before taking up Seddon’s invitation for a comparative philology, it is important to add that “drug” as an English noun goes beyond the boundaries of the English language. Indeed, the word instantiates a way of being in the world, a way through which multiple things are being thought. All languages are equal, but their projection of meaning is not.

What I mean by this re-use of Agamben’s suggestion? I mean that the extraordinary nature of the term “drug” in the English language, the fact that it encapsulates both “medicine” and “poison,” is what has come to matter in the making of the colonial and postcolonial politics around drugs.

**Figure 1:**
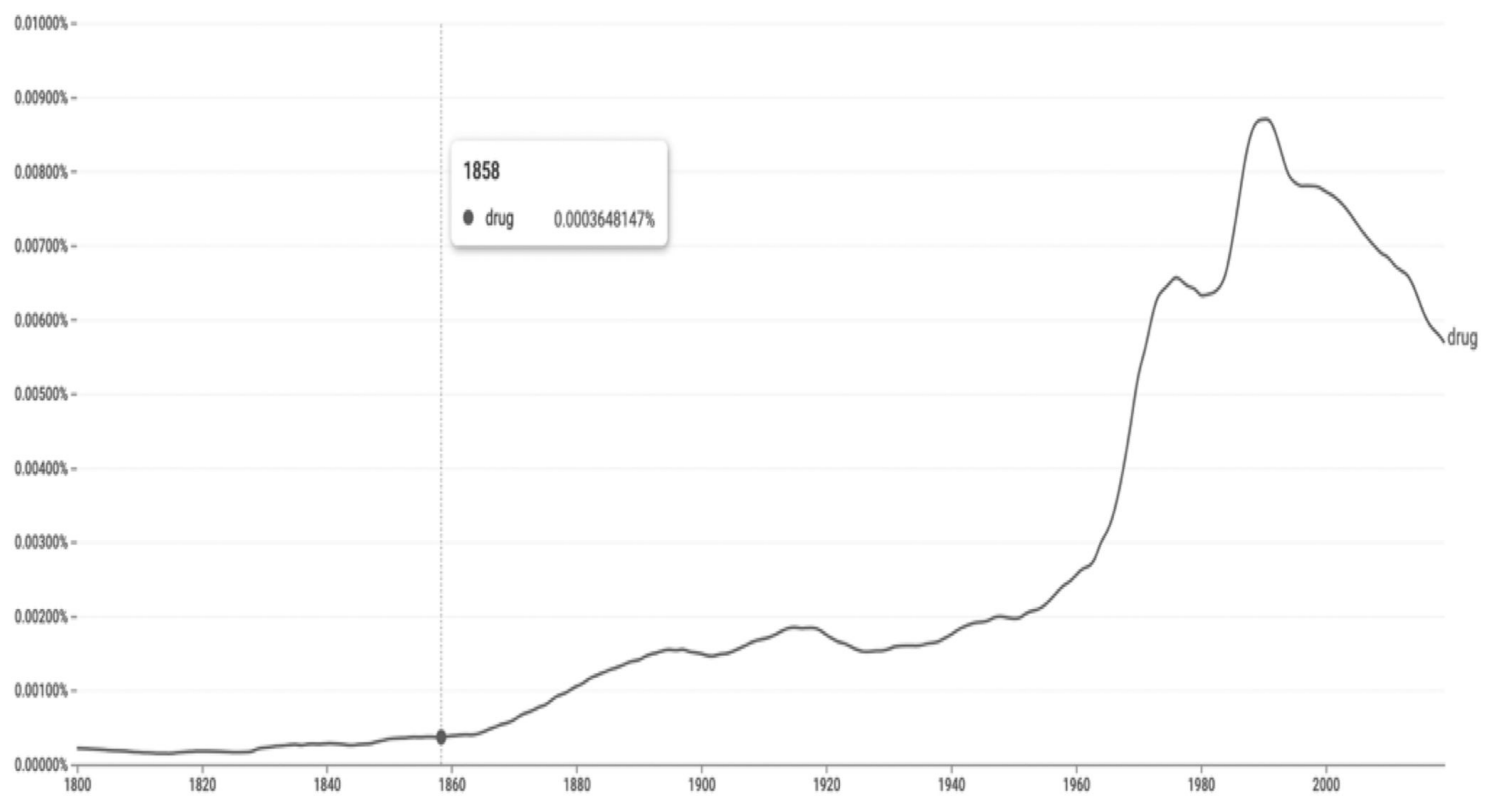
Ngram view of the word “drug” since the 1800s Source: Google Ngram Viewer, https://books.google.com/ngrams/graph?content = drug&year_start = 1800&year_end = 2019&corpus = 26&smoothing = 3&direct_url =tl%3B%2Cdrug%3B%2CcO, Color version available as an online enhancement.

From a historical viewpoint, “drug” is a medieval term that came into widespread use after the 1880s and, more vigorously, in the 1960s, possibly concomitantly with the treaty of the Single Convention on Narcotics Drugs (1961) (fig. 1).^25^ It found ample use in the colonial context—for alcohol in Ireland, ganja in Bengal, and bhang in Cairo, as reported in the *British Medical Journal*.^26^

In the Islamicate world, the Arabic and Persian equivalents for “drug” are *mokhaddir* (pl. *mokhaddirat*) and, in Farsi, *mavadd-e mokhadder* (narcotic substance).^27^ There is no equivalent, at least at face value, of the ambiguous term “drugs,” although elsewhere *taryak* is referred to as the *pharmakon* of the Islamicate world.^28^ From a historical viewpoint, *mokhaddir* came into use only following the institutionalization of the word “drug” in the official colonial and international discourse on control of mind-altering substances around the end of the nineteenth and the early twentieth centuries. Before that, mundane and scientific discussion had to make use of the real-world names of substances, devoid of the moral(istic) and pseudo-scientific connotations imbued in the term “drug”; however, the Orientalist Franz Rosenthal suggests that in pre-and early Islamic periods, *banj* (*bang*) might have been used as a generic (at times derogatory) term for all intoxicants.^29^

Terms vary conspicuously across the Islamicate world and travel across the Global South with nomadic erraticism. For opium, it is *taryak(q)* and *afyun*, both terms of ancient Greek origin, indigenized. For cannabis, the variation is broad and mutating depending on quality, make, typology, and places of origin—*shahdaneh*, *charas*, *hashish*, *ganja*, *esrar/asrar*, *sabzeh*, and *kif* are just a few of the transnational common words in use since the thirteenth century. In his study of medieval Arabic manuscripts relating to cannabis, Rosenthal admits it is not always easy to know from the nouns being used, what type of intoxicants are being referred to. Different trade names for “certain confections” and changing nomenclature in different localities with transient usage all suggested that cannabis had a dynamic existence across the Islamicate world.

The colonial adoption of the term “drug” performed an “epistemicide,” a caesura in local knowledge processes. As de Sousa Santos puts it, “unequal exchanges of knowledge among cultures have always implied the death of the knowledge of the subordinated culture, hence the death of the social groups that possessed it.”^30^ Colonial science on “drugs” granted to itself an epistemological privilege, buttressed by colonial enforcement of the evidence-based law or by the mimicking elites in new spaces of modernization and (nation) state formation. This resulted in the delegitimization and, later, dismissal of alternative knowledge and related social practices as performed by agents who were not aligned with coloniality/modernity.^31^

The case of alcohol is emblematic of how nomenclature shrinks when epistemicide is at work. A commodity like other mind-altering substances when it came to moral, legal, and social practices in the Islamicate world, alcohol has had a prominent, albeit often unrecognized, role in the history of Islamicate societies.^32^ The term “alcohol” derives from an Arabic word, *al-kuhl*; the Iranian lexicographer Muhammad Ibn Ya’qub Firuzabadi (1329-1415), who compiled the *al-Qamus*—one of the most comprehensive Arabic dictionaries—included 357 terms in reference to wine only. Moreover, the Arabic language has separate verbs to indicate drinking wine in the morning, afternoon, evening, and late at night (as well as other variations).^33^ We can infer something of the vibrant social life of alcohol—and intoxication more generally—through the mere existence of this vast lexicon, as already discussed for cannabis. Most of this nomenclature was lost with the arrival of colonial modernity and the institutionalization of religious law that came with it. Mahmood Mamdani referred to how enforcing tradition entrenched colonial powers, the latter being the first modern political fundamentalists claiming knowledge of an “original and pure tradition,” such as Islamic law, and the need “to return to that original condition” through the institution of the law.^34^

In the sphere of intoxication, epistemicide did not mean that Islamicate knowledge (Oriental) became outdated or weaker and thus supplanted by the more powerful and up-to-date Western “science.” In practice, most colonial authorities—including scientists—had patchy and haphazard knowledge of mind-altering substances in the colonies. This ignorance, however, was clothed in denial of value and validity of what colonists referred to as “local traditions” as opposed to modern science. The decoupling of local knowledge from colonial Western knowledge had a profound impact on sidelining and, eventually, burying indigenous knowledge of “drugs” in favor of the emerging sciences of medicine and pharmacy. This move was further legitimized by the upholding of philological knowledge about a time of origins (early Islamic history) in which prohibition of intoxication was (deemed) to be the rule/law.

Colonial history is rife with examples of ignorance clothed in knowledge. Philology and Orientalist histories played an important role in this production of ignorance. German missionaries in the 1850s believed, for example, that the word *dagga* as used in Southern Africa derived from the Arabic term for smoke, *dukkhan*. The justification for this presumed etymological link was the Orientalist myth of the intrinsic marriage of Muslims (and Islam) with cannabis culture rather than acknowledgment of the culturally integrative spaces of trade within the African, Arabian, and Indian network. Rather than the etymology being intrinsically erroneous, it is the logic of cultural essentialism voiding its historicity.^35^ In another example, the title of a postcard (dated 1911) portraying a group of Moroccan men smoking cannabis reports, “Un groupe de Hchachia (*fumeurs d’opium*),” confusing hashish (cannabis) with opium.^36^ Many other examples could be drawn from the annals of drug history.^37^

The outcome is an oxymoron: ignorance has been key to the production of knowledge about the term “drugs.”^38^ Philology has played its part—and that is why a decolonialization of drug histories should start from a reassessment of words and nouns. The best-known case of philological knowledge/ignorance in drug history is the trope of the *hashishiyin*—that is, the “Assassins.” Reassessing the story of the “Old Man of the Mountain,” written in Marco Polo’s *Il Milione*, the French Orientalist Antoine Isaac Silvestre de Sacy (1758-1838) gave scientific allure to a mythology around cannabis, linking the Arabic term *hashish* (“grass” or “cannabis”) to the etymology of the French term *assassin*. His argument was that cannabis-induced madness would compel the Nizari Islamili *fedayin* (“self-sacrificers”) to follow orders no matter how daring and dangerous, including assassinations of government and ruling officials. According to de Sacy, cannabis smoking and use mimicked madness and explained the roots of Muslims’ blind faithfulness to authority. Historian David Guba provides a detailed account of de Sacy’s argument and its global reach.^39^ The resonance of this mythology is hard to exaggerate. Debunked by historians and philologists from an early time, the myth has taken a life of its own, built on colonial ignorance with a tactical role in delegitimizing local epistemes of intoxication among the colonized and non-Western peoples.^40^

Ignorance, in the guise of mythologies, travels long epistemic distances and chains, contributing to intersecting theories about the non-Western world. In a 1932 text, the spread of Islam in Africa was still associated with the habit of cannabis smoking, “for some obscure reason—perhaps the prohibition of alcohol beverages.”^41^ But then alcohol bans and abstemiousness were the exception rather than the norm throughout the history of the Islamicate world, except for the period coinciding with the advent of colonial encroachment and modernity.^42^ The same largely applied to cannabis and opium (and other substances).^43^ It is true that one of the first national bans on cannabis was recorded in Egypt (in 1879)—the first of such bans being the British Empire’s ban on cannabis smoking for indentured laborers in Natal (1870)—and it was South Africa and Egypt that pressured the League of Nations into including an international cannabis ban.^44^ However, these and later prohibitions fell within processes of modernization and state formation in the advent of—or faced with the threat of—colonial modernity.^45^

Through this first epistemological step, I explored how people referred to different intoxicants (e.g., alcohol, cannabis, and opium) and asked what this use of words and names could have meant for them before the encounter with modern colonial knowledge and ignorance production. As an epistemic event, modernity/coloniality also occurred through nomenclature, which is where, I suggest, is possible to recognize the complexities of local knowledge before epistemicide. This step is vital if the aim is to liberate our understanding from the colonial legacy, with its totalizing force made of useful myths in the service of governance (in the colony on the colonized and in the metropole on the Other).

### Second step: Everyday Histories

Words on their own are not sufficient to decolonize drug histories. Philology and new vocabularies need to be put into practice and into new interpretative historical frames. My proposal—which does not promise to be exclusive or all-sufficient—is for drug history to lower the gaze to the everyday and move back to ordinary occurrences and settings rather than to the panopticism of colonial and colonized institutions (e.g., League of Nations, psychiatric hospitals, prisons) and their archives. Beyond conventions, laws, and regulatory regimes stands a vibrant historical phenomenology.

Nevertheless, this is easier said than done, given that consumers of mind-altering substances become visible especially when they turn into identifiable problems: crime, alleged madness, poverty, and health hazards or objects of exoticism. To bypass these analytical obstacles, historians can look for people’s practices to put drugs beyond a colonial and colonizing gaze. This requires anthropological sensitivity to the historical material that can be facilitated by asking the right questions rather than by changing the tools of the craft. For instance, how can we unpack people’s religious life when linked to the use of drugs? What are their aspirations in engaging with mind-altering substances? How did they use drugs in work and nonwork activities, love, hatred, emotions, social conditions, and so forth.?^46^

Seen through these questions, the everyday is not a field but a perspective. Its “focus is on the forms in which people have ‘appropriated’—while simultaneously transforming—’their’ world.”^47^ In the Islamicate world, there are numerous scientists and medical professionals who dealt with the uses of mind-altering substances—and ways to minimize the harms—in everyday life. I provide a brief discussion of a medical treaty known as *Resaleh*-*ye Afyuniyeh* (The Opium Treaty) by the physician ‘Emad al-Din Mahmud Bin Mas’ud Shirazi (died 1515).^48^ One telling example drawn from the treaty refers to the prescription of a “slow-release opium suppository” and “pill” for opium eaters who were struggling to comply with the religious requirements of fasting during the holy month of Ramadan, when Muslims should refrain from drinking and eating from sunrise to sunset.

The physician divided opium users according to their daily intake (one, two, or three or more times daily). He advised those consuming twice daily to plan ahead of the month of fasting by changing their time of intake in the months of Rajab and Sha’ban (the two months preceding Ramadan) so that they could stretch the time between the first and second intakes. In this way, they would be able to use opium right before sunrise and right after sunset. Instead, the slow-release suppository and pill provided a remedy to be used only by those who could not adapt by changing their time of intake. In another section, the physician identified the rationale behind opium addiction as caused by pain and misery resulting from great changes in people’s everyday lives due to socioeconomic factors caused by dramatic political change. He wrote, “They have recourse to opium in order to suppress their distress.”^49^

This treaty is revealing of a radical epistemological approach toward intoxication, addiction, and health. It regarded addiction as a life condition requiring practical understanding and adjustment rather than religious condemnation, persistent medical intrusion or—worse—legal prosecution. This notion of addiction, moreover, was in stark contrast with the modern West-centric concept as a moral failing, criminal offence, disability, or source of risk, as it had been since its late nineteenth-century appearance.^50^ Considering Shirazi’sinfluence over successive generations of physicians and scholars reflecting on and practicing in opiate use, one can see that this treaty represented a form of *ante tempore* harm reduction for people consuming opium in their everyday life.

Ramadan is a moment of heightened tension and significance in matters of intoxication.^51^ The end of the fasting days had historically been animated by street vendors and charitable groups offering “water and opium *[ab-o taryak]”* to all those in need.^52^ This practice remained until the mid-twentieth century, together with other practices supporting opium users. As an example from everyday parlance in the twentieth century, places designed for smoking *shireh* were known as *dar al*-’*alaj*, meaning “clinic” or “house of cure.” They provided comfort for those who, having developed a chronic habit, could find a safe space to use opiates and be functional members of society. Supported and recognized as having a social utility and public function, these clinics were nonetheless targets of criticism and moral outcry by groups of city dwellers.^53^ Contention and nonconformity to legal disposition has been an enduring feature of the lifeworld of intoxication in Iran.

A parallel example supports this claim. During the first Pahlavi period (1925-41), itinerant buses drove across the southern parts of the Iranian capital Tehran—the areas surrounding the Great Bazaar—offering support for opiate users in need. Mindful of modern harm reduction outreach teams (but more radical in their pursuit), a contemporary account reported that the bus driver’s assistants would shout, *mordé mibarim, zendé miyarim*: “We take the dead away, we bring them back alive.”^54^ The provision of care distinguishes public, although not state-led, interventions for opiate consumers, implicitly acknowledging that for those with a chronic habit, the safe provision of the substance supports improvement in health and social life.

The question of agency and the “everyday” should not be misinterpreted as an inherent force against coloniality/modernity. Agency can often perpetuate modernization or be instrumental to it. The claim that the everyday life of “drugs” was entrenched in people’s existence in the Islamicate world should not suggest that cannabis and opium had fixed and normative places in these societies. Reformers and revolutionaries across the Islamicate world associated cannabis and opium with decadence and an inability to progress to the standards of modernity. During the 1905-11 Iranian Constitutional Revolution (*Enqelab-e Mashruteh*), one of the first undertakings of the revolutionaries after entering triumphantly in Tehran was to attack the opium smoking houses and collect all (working class and poorer) people perceived as *taryaki* (denigrative word for opium “addicts”). It was with this spirit that Iran took part in the first Opium Conventions in Shanghai (1909) and The Hague (1912); with reformist momentum, the constitutional government—weakened by internal divisions and pressures from imperialist states (Russia and Britain)—introduced the first laws limiting opium consumption and providing a seven-year period for those deemed “addicted” to kick their habit.^55^

The ideological association between reformist and revolutionary politics and antidrug campaigns is known and well discussed in annals of drug history.^56^ The same goes for indigenous conservatism allied with modernist elites and their pursuit of local bans.^57^ In the case of the Is-lamicate world, Islamic jurists such as the Damascene scholar Ibn Tay-miyya (died 1328) invited uncompromising measures against the use of cannabis, comparing it to “wine drinking or worse.”^58^ Islamicate countries (and much of the colonial world and the Global South) were not passive recipients of drug control and/or modernization. Fear of novel products and social habits as well as contending opinions about what is intoxication and how society should regulate it, animated the debates about consumption of substances, including the case of a coffee that was met with hostility by various groups in both the Ottoman and Safavid empires.^59^

With the coming of age of the modernizing state, local institutions acquired far-reaching force in making knowledge. They invested in colonial knowledge frames on “drugs,” situated in local everyday realities. However, this projection did not successfully eradicate alternative drug lifeworlds, which maintained and adapted their significance in everyday practice. Indeed, tension between agency and modernization has been a driving force in everyday drug history, as exemplified in the experimentations of regulations, restrictions, prohibitions in the first half of the twentieth century, medicalizing regulations again in the 1970s, and then with the Islamic Republic’s harm reduction policy since the 2000s.

An example of such initial tensions was the petition to the Iranian Ministry of Finance, signed by the self-proclaimed “70,000 addicts of Abadan [*mo’tadan-e Abadan*]” on 10 October 1941, which captured this tension in idiosyncratic ways:

With all due respect to your excellence, we address you on behalf of the 170,000 inhabitants of the city of Abadan, at least 70,000 of whom are drug addicts. In recent days, the opium distribution branches in the city have been closed by the Taxation Office. Of the twenty-four branches in this city, only one remains. In addition, this branch only gives half a gram of opium to each addict. We are sure you will easily understand that the decrease from five grams per day to half a gram causes extreme physical weakness that will prevent people from working, and consequently destroy the large families whose resources depend on these workers.^60^

This document is interesting in many respects. A group of local citizens of Abadan addressed a modernizing state institution led by an American civil servant, Dr. Arthur Millspaugh. The petition used a language and terminology that, to a foreign reader, may sound exclusively denigrative and delegitimizing (e.g., “addict”). However, the document cannot be read exclusively in this way. By reclaiming the term “addict” as *mo’tad* (neutral) and not *taryaki* (negative), the petitioners were mobilizing an implicit script that traces its genealogy to the local historical experience of opium and opiates, as shown in Shirazi’s medical treaty on opium.^61^ They were proposing an alternative understanding of addiction—and thus opium consumption—counter to the modes advised and imposed by Western medical science and pseudo-colonialist administrations. In so doing, the petitioners reclaimed a form of life and a lifeworld that stood outside coloniality/modernity—one in which opium was a right to those who needed it to prevent “extreme physical weakness,” rather than causing that weakness in the first place.

The letter also referred to environmental conditions as worthy of consideration when thinking about “addicts” and opium users; this was an invitation to reflect on opium lifeworlds beyond coloniality/modernity. The petitioners reminded the American administrator that the city of Abadan is in a geographical area with a “humid climate,” which increased bodily and joint pains. Notorious for its unwelcoming conditions, Abadan is close to the swamps of the Arvand Rud river (Shatt al-Arab) and the southern coast of the Persian Gulf. Following Sir Percy Cox’s success in negotiating the Anglo-Persian Agreement (1919), Abadan became the epicenter of the British expansion in the oil industry. The surrounding region of Khuzestan is home to the world’s largest oil reserves, themselves an epitome of modernity/coloniality and the main driver to capitalist modernization, in Iran and globally.^62^ Seen from the “everyday” perspective, the oil industry meant “exhausting work in the Anglo-Iranian Corporation,” which brought thousands of people to the region under working conditions that were harsh and debilitating.

Millspaugh’s response did not fall short of colonial expectations. In a short note, he said that “according to the regulations in force . . . opium consumption must gradually decrease. Opium rationing meets this objective, with the municipal tax office responsible for distributing half a gram to opium eaters and two grams to those who smoke it. Thus, the complaints of drug addicts have no rational justification.”^63^ Irrationality equates with both the colonial view of the Orient and the colonized habit of “addiction.” The request for provision of health conditions in the otherwise inhumane working environment of the oil fields was dismissed without much fanfare. The progressive eradication of the opium habit was ideologically enshrined in the state’s modernizing push and endorsed by the economic and cultural elites, who, even when consuming opium themselves, saw the habit as unfitting to the poor and the worker.^64^

The rupture in the ecology of knowledge and its everyday practices on intoxication came with the Pahlavi government’s adoption of opium prohibition laws in 1955.^65^ This law represented the first instance of a comprehensive policy to eradicate poppy cultivation from the Iranian plateau; its implementation followed the rise of US influence over Iranian polity in the wake of the 1953 coup against premier Mohammad Mossadegh.^66^ When the opium ban was eventually approved, the decision was justified by “modernisation,” in the words of the then Minister of Health, “which in most developing countries means imitation of Western models and abandoning’ a dark Oriental past.”^67^ This statement itself shows how the question of opium was perceived by the Iranian ruling elites as an issue of modernity/coloniality. The initial draft of the law, redacted under the lobbying pressure of the Society for the Fight against Alcohol and Opium, had included a total ban on alcohol consumption together with opium. However, any reference to alcohol was eventually dropped. Alcohol was a hallmark of Western civilization, and Iranian modernity had to come with Western habits.

### Lifeworld Policy Alternatives

History and policy do not follow linear evolutions. Knowledge of localized practices and their genealogical scripts can reemerge and transform policy. By 1969, what represented an adoption of colonial policies on intoxication on the part of Iranian modernizers was turned around. A national policy of opium maintenance made opium available to all registered users through the pharmacy and physicians, in line with genealogical recipes such as the one articulated by the physician Shirazi. Rather than a revival of the “British system” of heroin maintenance, this new policy emerged from an attempt to disentangle and extricate domestic policy from the script of Western prohibitionist dogma.^68^ It also responded to the impossibility of eradicating the transnational trafficking networks originating in Afghanistan. It became an ontological policy that transformed the place of opium and put it beyond Westcentric scripts of biomedicine. “Care” for consumers and social place in everyday life informed the policy, as opposed to Western scientific prohibition of drugs and prevention of addiction. The West’s primary concern when it came to opium—and other drugs—was not scientific or health related but rather security oriented. It was a concern governed by colonial/imperial rationale rather than international collaboration. The president of the International Board of Narcotics Control (INCB), the prime body representing Western interests in matters of drugs, summarized this aspect well. For him, Iran’s policy was lacking “wisdom” and was not fit for purpose in countering “the illicit supplies of opium which were coming into the country.”^69^ His advice was for tougher and stronger policing and anti-drug trafficking in addition to opium prohibition. History teaches us that this is the wrong recipe.

Beyond the INCB’s concern lies the allure of a scientific confrontation between science on intoxication informed by localized medical histories and Western colonial knowledge dismissive of other practices. This was exemplified by the diatribe occurring between Iranian scientists and their Western counterparts. Meeting in Ramsar for the Central Treaty Organization-sponsored conference on international public health, Western scientists accused Iran of going backward and against the countries’ modernization program, which was most notably embodied by its embrace of opium prohibition. In his welcome address, Dr. Hasan Morshed, Iran’s undersecretary for parliamentary affairs of the Ministry of Health, declared that the subject of the meeting was “very important to us in view of the fact that the Government of Iran looked on addicts *as real patients*.”^70^ To put it in perspective with the philological discussion outlined earlier, the “addiction” of premodern Iranian science held no moral outrage in its etymology, as opposed to the etymology of Western “addiction.”

Similar to what was demonstrated by Isaac Campos’sworkonMexico, ideas about control and punishment of mind-altering substances were integral part of the political grammar of these societies.^71^ Institutionalization of repressive control regimes on intoxicants came as part of a historical process in which the agency of indigenous forces produced effects more colonial that colonial powers themselves. Local modernizers dismissed historical knowledge, genealogical scripts, and the plurality of ethical orders around social practice and the everyday, leaving little room for ambiguity in the defining “drugs” or “intoxication.”

The work of decolonizing histories—and drug histories, especially—must be conscious of the need for demystification, starting from the lexicon of “drugs” and moving to “everyday life,” where the law, coloniality/modernity, and agency can be seen in practice. Beyond the image/word of “drugs” lies entangled histories of capitalism, racism, colonial modernity, and radical alternatives to these historical forces. In this regard, drug histories have the potential to be entangled histories of the present amid the ruin left of modernity/coloniality.

In this second epistemological step, I uncovered the links between medieval, early modern, and modern lives of intoxicants from the Persianate and Islamicate worlds. Rather than unflinching prohibition, this history reflected the concern about the everyday in the lifeworld of intoxication, both in reducing negative health effects and in debating regulatory measures before the monochromatism of Western modern science on intoxicants.

### Third Step: Reclaiming Heterodox Histories

When it comes to drug histories, time and narrative are two defining features of everyday life. On one hand, there is the rhythmic repetition of events punctuating the everyday, when uncovering the habits of consumers or those seeking care or help; on the other hand, there is a radical alterity and, indeed, illegibility of people under the effect of mind-altering substances. Their lifeworld is harder to read because it often breaks the boundaries of social norms and sits at odds with ordinary life. So far, this article has provided examples from premodern and modern history, involving medical treaties, petitions, regulatory texts, and historical observations of everyday life. Now I invite you to see the radical alterity of Islamicate drug histories by focusing on a lost historical and literary figure: the self-proclaimed “intoxicated,” known as the *rend*.^72^ Siraj Ahmed, in his reading of the Iranian poet Hafez (1315-90) and his influence throughout the Islamicate world, invoked these historical and literary figures as “a critique of the very complex that would eventually align itself with European colonial rule.”^73^ In different variations (*rend, qalandar*, *malamati*), these terms referred to the quintessential noncon-formist heterodox individuals who came to occupy a special place in public and literary imagination starting from the fourteenth century. Their distinctive feature was the embrace of a form of life of public disobedience against religion, capital, and power. This included unapologetic and at times confrontational intoxication with whatever the law of the land or the Islamic jurists (*faqihi/foqaha*’, ‘*alem/*’*olama*’, *qazi/qozza*’)declared to be forbidden, be it wine, cannabis, or opium or heteronormative sex. It also included embracing poverty as nonjudgmental existentialism in the name of love for life and God. Rather than being solitary mystics (*zahed/zahedan*) who preach world abandonment, the *rend* lived outside the law (and, in the refusal to write under an official name, of history).^74^ This radical alterity makes the *rend* an epistemic paradigm, part and parcel to the experiment in reimagining drug history outside West-centric scripts from within the Islamicate world.

In the *rend’s* ethics of life, salvation and the pursuit of a life worthy of divine creation depended on one’s capacity to reject human hierarchies and conventions. This could not happen through asceticism, as imagined in Orientalist scholarship, but rather through “ceaseless confrontation with [ruling] culture: [these figures] self-consciously created a ‘social wilderness’ within society.”^75^ In the ontological rather than metaphorical sense, life could only be called as such beyond the law—life destitute of all conventions.^76^ What I suggest is to look at these figures “of counterculture and disrepute,” who lived in “ruins [*kharabat*]“ animated by wine drinkers and cannabis eaters and their lovers, with the purpose of reimagining the Islamicate lifeworld of intoxication beyond colonial gaze but through the poesies of those who were themselves intoxicated. This works as a methodological displacement from official scripts produced by Western observers and Orientalists, even when, as in the case of Rosenthal, they were profoundly knowledgeable of Islamic textual histories.

## II

An enduring question has animated the reading of Hafez’s poetry (and other Iranian poets with a mystical or ethical orientation): were his words symbols reflecting an ethical reality, or was he reflecting on “a transformative realism”—a social history through universal figures of non-conformity embodied by the *rend*, the drunkards, the intoxicated, and antinomian outcasts living beyond the law and history?^77^

It is beyond the remit of this article to respond to that question. But it has been ascertained that intoxication had substantial influence over the social and spiritual lives of people of different classes and cultural backgrounds, starting from the period of Hafez’s poetry and through the latter’s influence in the Islamicate world—for instance, in the Indian subcontinent and Turkic world.^78^ Through Hafez and other like-minded thinkers, we can work toward reclaiming an ontology of drugs from the South, outside West-centric scripts of medicine, pleasure, and poison. In the words of the poet:

فقیه مدرسه دی مست بود و فتوی دادکه می
حرام ولی به ز مال اوقاف است

Yesterday, the jurist in the school was drunk; he launched a *fatwa* [religious decree]:/

Wine is prohibited [*haram*], but it is still better than all religious endowments.^79^

The jurist in the school, himself drunk, understands that even though wine (and, by extension, all intoxication) will always remain beyond legal acceptability for Islam as a religion (and as public order), drinking and being intoxicated are surely more worthy than the hypocrisy of those who condemn it. This permanently fleeing ethics of intoxication and drunkenness could provide drug histories with the epistemic force to move beyond coloniality/modernity and decoloniality and toward new spaces of writing people and substances into the knowledge of the world. As ontology, it leaves the lifeworld of drugs in a fluid uncertainty on which judgment remains wanting and, by necessity, inadequate.

Next, I provide several examples of how the figure of the *rend*, and its equivalents, displaced dichotomic ethics around mind-altering substances, including redrawing the boundary between alcohol and opium, therapy and pleasure, and knowledge and intoxication.^80^

منم که شهره شهرم به عشق ورزیدنمنم که به
دیده نیالودهام بد دیدنوفا کنیم و
ملامت کشیم و خوش باشیمکه در طریقت
ما کافریست رنجیدنبه پیر میکده
گفتم که چیست راه نجاتبخواست
جام می و گفت عیب پوشیدن
...
به می پرستی از آن نقش خود زدم بر
آبکه تا خراب کنم نقش خود پرستیدن
...
عنان به میکده خواهیم تافت زین مجلسکه
وعظ بی عملان واجب است نشنیدن
...
که وعظ بی عملان واجب است نشنیدن مبوس جز لب ساقی و جام
می حافظکه دست زهدفروشان خطاست بوسیدن

I am the one famous in town for love-making / I am the one who’s not bothered with bad seeing.

Let’s be faithful, endure blame and be happy / For in our path to suffer is blasphemy.

I asked the old man of the tavern, “What is the road to salvation?” / He demanded the wine cup and said, “To cover one’s fault.”

I submitted my fate to wine-worshipping / To destroy the fate of self-worshipping.

Riding away from this religious assembly, we will gallop to the tavern /

For one must not listen to the preaching of those who do not practice [intoxication].

Hafez, do not kiss anything but the cupbearer’s lip and the wine cup / For it is a mistake to kiss the hand those preaching world abandonment.^81^

Hafez clothes his poetic person in that of a rend.Hepursues “love” and leaves judgment and criticism to others, who abide by mundane conventions. His pledge, which comes almost in the form of a prayer, is to embrace the blame by the rule makers and abiders because the only thing that matters in life/history is the pursuit of happiness beyond the self. Indeed, the poet here uses the religiously charged word of *kafar* (blasphemy) for those who refuse this form of life. (Blasphemy being punished with death in Islamic law, unless judged as caused by madness, for which there is no punishment but a downgrading of the individual to the status of a minor). If we read judgment as being the prerogative of coloniality/modernity, then the poet’s question about “the road to salvation” parallels the colonized wondering about how to be modern. The old man of the tavern, who symbolizes those who have reached spiritual wisdom and refused conventions, responds that the only salvation is to be open about one’s faults and shortcomings. Hearing that, the poet understands that wine worshipping—that is, freedom from orthodoxies and imitation models—is the only way to go beyond the law or worshipping the self, of pursuit coloniality/modernity. That is how he rides away from the *majles*, “the religious assembly,” toward the tavern, because one cannot learn how to change from those who do not indulge in life. The religious assemblies spend time discussing prohibitions and the harms of drinking and intoxication, but they are those who do not take part in drinking and intoxication; there is no value in listening to them because they lack knowledge/practice. In the same way, colonial modernity’s refusal to take seriously the science of intoxication developed in the Islamicate world is not worth listening to, if not as a form of ignorance/power. Indeed, those who preach modernization and abandonment of that which pertains to one’s way of life cannot be thanked: the poet refuses to kiss the cup of orthodox modernization.

This decolonial interpretation of Hafez’s poetry may sound forced, but the times of rejection of orthodoxy, embracing intoxication as a form of life, the questioning of knowledge/ignorance are recurrent critical tropes in his poetry. In another poem, Hafez refers to opium and to those who are “in pain” and the need to listen to them when making sense of the world—we could say, when writing (drugs) history:

از آن افیون که ساقی در می افکندحریفان
را نه سر ماند نه دستار
...
بیا و حال اهل درد بشنوبه
لفظ اندک و معنی بسیار

That opium which the cup-bearer dropped in the wine-cup / Left neither head nor turban to the rivals.

...

Come and listen to the state of the people of pain / With few words and much meaning.^82^

The mixture of opium and wine hints at the breaking of the two dimensions of religiously prohibited substances (wine) and those substances that are seen as part of the everyday life in ambiguous ways (opium.) There is no value in this pretended division, as they both (and, indeed, together) displace the authority (turbans) and the minds of the lawmakers and the power holders (*harifan*). Instead, to pursue meaning, one must listen to “the people of pain” (*ahl-e dard*), who, we may hypothesize, cure their state with opium and wine.

## III

The *rend* was a figure, both historical and metaphorical. It was a name with ontological force. Through this figure, embraced by the poet, it was possible to reimagine an epistemology of intoxication around substances in indigenized terms. The poet reflected on the experience of intoxication in the face of orthodox condemnation, legal and moral orders, and the hypocrisy of those in power.^83^ Rather than a didactic, linear exposition, this method aimed to convey “feel-thought” (in Spanish *sentirpensar* as enunciated by Orlando Fals Borda)—”a way of knowing that does not separate thinking and feeling, reason from emotion, knowledge from caring.”^84^ In itself, feel-thought is reminiscent of the practice of mystical (but not ascetic) orders, not unlike the ones mentioned, which pursued *ilm al*-*dhawq* (the science of taste) in discerning the world: only direct experience—tasting—of the world can lead to knowledge, even when in stark contrast to religious commandments.^85^ This approach, it-self deserving a separate treatment around transcultural alternatives to West-centric methods/theories, links Islamicate heterodox practice with the emerging decolonial epistemologies of the South (especially in Afro/Latino America, Abaya Yala).^86^ As a prelude, it could work to rearrange knowledge in writing drug histories in the Islamicate world.

Other intellectual figures embraced this feel-thought approach to the science of intoxication without recurring metaphorical poesies but rather by direct invocation of their listeners. A contemporary of Hafez, the poet and satirist Obeyd-e Zakani (1300-1371), was the emblematic *rend* of his epoch. His spirited descriptions of everyday life in Shiraz were notorious for their depictions of intoxication and sexual promiscuity and critique of orthodox hypocrisy. A satirical short story about a worshipper cooking cannabis in the local mosque provides an example: Faced with the mosque custodian’s reprimand, Obeyd wrote, “the Shirazi looked carefully at [the custodian] and saw that he was extremely ugly. He was [also] lame, deaf, bald, and blind. The Shirazi responded by saying, You inferior man! Is it because God has not been very kind to you that you are driven to be so overtly prejudiced about his house?”^87^ The satirist implies that prohibitions are man-made, whereas pleasures are worthy even inside the place of worship. In another poem, he evokes the life of a very intoxicated person:

سحرگهان چو ز خواب خمار برخیزیخیال
بنگ و نشاط شراب خوش باشد

In the morning when you awake from sleep hangover / may you still enjoy cannabis’s dreams and wine’s joy.^88^

A commonsensical and overt pledge to the pleasures of intoxication are how the satirist contrasts the authority of the law. As a *rend*, he must act without preaching, “for one must not listen to the preaching of those who do not practice,” to quote Hafez’s earlier poem.^89^ This ethics of intoxication traveled well into the twentieth century. The tension is captured by Parvin E’tesami (1907-41) in her poem, *The Drunk and the Sober* (*Mast va Hushyar*), a dialogue between an intoxicated passerby and the policeman who stopped them:

محتسب، مستی به ره دید
و گریبانش گرفتمست گفت ای
دوست، این پیراهن است،
افسار نیستگفت: مستی،
زان سبب افتان و خیزان
میرویگفت: جرم راه رفتن
نیست، ره هموار نیستگفت: میباید
تو را تا خانه قاضی برمگفت: رو صبح
آی، قاضی نیمهشب بیدار نیستگفت: نزدیک
است والی را سرای، آنجا شویمگفت: والی از
کجا در خانه خمار نیستگفت: تا داروغه را گوئیم،
در مسجد بخوابگفت: مسجد خوابگاه مردم بدکار
نیستگفت: دیناری بده پنهان و
خود را وارهانگفت: کار شرع، کار درهم و دینار نیست
...
گفت: باید حد زند هشیار مردم، مست راگفت: هشیاری
بیار، اینجا کسی هشیار نیست

A policeman saw a drunk (*mastī*) on the road and held their shirt collar.

The drunk said, “Oh friend, this is a shirt, it’s not a bridle.”

He said, “You are drunk and that’s why you walk falling and rising.”

The drunk said, “It is not a crime to walk, the road is not smooth.”

He said, “I must take you to the judge’s house.”

The drunk said, “I will go in the morning, the judge is not awake in the middle of the night.”

He said, “The governor lives nearby, let’s head there.”

The drunk said, “How do you know the governor is not hungover (*khomar*) in his home?”

He said, “To the sheriff, let’s go and sleep in the mosque.”

The drunk said, “The mosque is no dormitory for those committing bad acts.”

He said, “Give me some money and go away.”

The drunk said, “The work of the law is not the work of dinars and money.”

...

He said, “Aren’t you aware that your hat has fallen from your head?” The drunk said, “Reason must be on one’s head, there is no shame in being without a hat.”

He said, “Sober people should severely punish the drunk.”

The drunk said, “Bring me someone sober, here none is such.”^90^

In the debate between these two, emblematic of lawbreaker and law enforcer, it is the drunk showing intelligence inclusive of human experience and awareness, whereas the policeman follows reason and rationality but always falls short of understanding the ethics of life.^91^ It is a metaphor well suited to the contention between localized knowledge of intoxication and the modern colonial science of drugs. If expanded and pursued, these forms of life and their ethics may open novel, epistemological possibilities for the writing of drug histories in the Islamicate world.

In this third—and, for now, final—epistemological step, I introduced into the writing of drug history the *rend*, a historical figure that embodies intoxication as a form of life and, at the same time, shows how intoxication is an ungraspable human experience beyond religion, morality, and the law. This is what I referred to in the introduction as “ethics beyond morality” and “politics beyond society.” With this, I claim that the historical figure of the antinomian mystic, with their outwardly heterodox public presence, and the intoxicated person (*mast*), more generally, not only represent a critique of stigmatization but also affirm intoxication as a bearer of meaning and possibility in ethics, politics, and life.

## IV

Decolonizing implies decommissioning old and obsolete pedagogies.^92^ To redraw a decolonial history of intoxication, one cannot simply and exclusively rely on West-centric scripts and methodologies. There is need for further reflection on the tools that have so far informed drug history writing and openness about novel epistemological intrusions. That said, this article does not advocate dismissal of the existing historiography, much of which has undertaken a geocultural overture toward new spaces, and reassessment of the ethical coordinates informing drugs (and, less so, alcohol) in history in the past two decades. This shift in new drug history has instinctively or unconsciously taken up Fanon’s dictum that decolonizing is rearranging the space, including the epistemic space.^93^ Nevertheless, if the objective is an actual decolonial history, then its writing must also be open about and informed by epistemological contaminations and osmosis between and across theories of the West, South, and East, as much as intermediated by different disciplines reflecting on intoxication, health, and life.^94^ Ideas, concepts, frames, and formulas coming from the Islamicate world should be part of this epistemic journey. This approach may unsettle the historiographic flow, sitting at odds with the vocabulary and norms of the discipline or even with “interdisciplinarity.” But it may also hold the seed of transformative knowledge, by being “undisciplined” or transdisciplinary—by its being a *rendi* epistemology, an epistemology beyond our episteme. Here we might find the seed for decolonizing histories beyond and without the West.

In the Islamicate world, one needs to build a new epistemological frame on the ruins of Orientalist and colonial ignorance production. Thankfully, the work of historians in dismantling colonial ignorance production about the lifeworld of drugs has been strong and systematic. Now, as I argue and experiment in this article, the work concerns building on these ruins, within the ruins—the *kharabat* where the *rendi* gathered and gather.

One place to start this world-building history is through the reacquisition of a novel nomenclature displaced by the colonial/Western names, as enunciated in the first epistemological step of the article. Given their entrenched history and the embeddedness in knowledge/power through regulatory, scientific, ethical, and economic systems, historians must come to terms with the category of “drugs” and problematize the use of words as much as their implications in colonial practice and modern scientific knowledge and policy. A critical engagement need not to abandon the term necessarily, as “drug” itself is entering a phase of reassessment and reappropriation that reflects its ontological pluriverse, which is historically and epistemologically ambiguous.^95^

In his article collection on *Pluriversal Politics*, Arturo Escobar reflects on how to think and come up with other “possibles” and other “reals” as ways of conceiving life (and, we could add, history) otherwise. He finds inspiration in Latino American, matriarchal, and Buddhist philosophical thought, concluding that “derealisation means recommunalizing, reconnecting, relocalizing, deindividualizing ... [which could] lead us to attempt to leave ... a belief in one reality, one world, one truth, and ultimately one very blinkered vision of the possible.”^96^ This invitation is similar to the spirit of Hafez’s poetry, which I refer to as *rendi epistemology*, captured in a couplet noted earlier: “I submitted my fate to wine-worshipping / To destroy the fate of self-worshipping.”

Escobar’s and others’ pursuit of new epistemological visions of the world rests on engagement with indigeneity and critical thought across Latin America and East Asia, but it rarely recognizes the possibility of radical alterity and ontological potential in the Islamicate world. One of the purposes of this article was to experiment with epistemological possibilities in historical analysis from the starting point of the Islamicate world. This experiment was not carried out in isolation but rather as a form of cross-contamination in which, similar to the conventional boundaries of “drugs” and “medicine” or “pleasure” and “therapy,” ontologies flow across space/time carrying ideas and forms of life in their journeys.

In pursuing other epistemologies and in writing history of other ontologies of intoxication, I am thinking of de Sousa Santos’s motto that we are *facing modern problems for which there are no longer modern solutions*. The contemporary so-called drug or addiction problem cannot be addressed by the existing West-centric epistemologies alone; what is needed is a feel-thought, or *dhawq*, that can move human understanding of addiction and drugs beyond coloniality/modernity and their governing machine—capitalism.^97^ Although it is an exercise in understanding addiction and intoxication, this attempt may also shape the way in which we might reconfigure the experience of addiction and intoxication based on acknowledgment of their pluriversal possibilities, some of which may be life-enhancing—fulfilling of intimate desires and with political force.

It is through this passage that I attempt to move from studies of drug globalization and global drug studies to a new approach centered on the pluriverse of intoxication “as made up of a multiplicity of mutually entangled and co-constituting but distinct worlds.”^98^

This article is one step toward a conscious formulation of this pluriversal approach.

## Footnotes

1 Moulana Jalal al-Din (Rumi), “‘Maulana’ Divan-e Shams: Poems” (in Persian), *ghazal* (poem) no. 245, https://ganjoor.net/moulavi/shams/ghazalsh/. The text of poems cited in this article were consulted on ganjoor.net (in Persian) unless indicated otherwise. Persian and Arabic text is provided without diacritical transliteration.

2 Carlo Ginzburg, “On the European (Re)Discovery of Shamans,” *London Review of Books* 15, no. 2 (28 January 1993), https://www.lrb.co.uk/the-paper/v15/n02/carlo-ginzburg/the-european-re-discovery-of-the-shamans.

3 Boaventura de Sousa Santos, *Epistemologies of the South: Justice against Epistemicide* (London: Routledge, 2015), viii.

4 The Islamicate world has been marginal to the new wave of critical thinking and decolonial theory, as opposed to Latin America, the Indian subcontinent, and, recently, Africa, where intellectual sympathy toward localized manifestations of critique have been strong and celebrated in academia; see Walter D. Mignolo and Catherine E. Walsh, *On De-coloniality* (Durham, NC: Duke University Press, 2018); Achille Mbembe, *On the Postcolony* (Berkeley: University of California Press, 2001); Achille Mbembe, *Necropolitics* (Durham, NC: Duke University Press, 2019); Achille Mbembe, *Out of the Dark Night* (New York: Columbia University Press, 2021); Arturo Escobar, *PluriversalPolitics* (Durham, NC: DukeUniversity Press, 2020); Ashish Kothari, Ariel Salleh, Arturo Escobar, Federico Demaria, and Alberto Acosta, eds., *Pluriverse: A Post-development Dictionary* (New York: Columbia University Press, 2019).

5 The “Islamicate world” refers to the influence and presence (past and present) of Muslim communities across the globe in the form of overt or implicit cultural, social, religious, and practical imprint. A term parallel to “Islamicate” is “Persianate,” which refers to the influence of Iranian and Persian cultures beyond the remit ofIran-based empires and power formations. The terms were coined by historian Marshall Hodgson to capture the civilizational scope of Islam beyond its often overstated religious semiotics (for Islamicate) and ethnic categorization (Persianate). Persianate, however, overlaps with the fluid notion of an “Iranian world”—not to be confused for the nation-state—which refers to the geocultural space where Farsi and other Iranic languages (Kurdish, Taleshi, Baluch, etc.) were dominant and where Farsi was the lingua franca of the time (in literature and administration): West and Central Asia and the Indian subcontinent.

6 Edward W. Said, quoted in Joseph A. Massad, *Islam in Liberalism* (Chicago: University of Chicago Press, 2015), 2.

7 See Toby Seddon, *A History of Drugs: Drugs and Freedom in the Liberal Age* (London: Routledge, 2010).

8 Mignolo and Walsh, *On Decoloniality*, 4.

9 For colonialism and race and ways to think beyond them, see Mbembe, *On the Postcolony*.

10 For drugs and race, see, e.g., Kojo Koram, ed., *The War on Drugs and the Global Colour Line* (London: Pluto, 2019); Alexander S. Dawson, *The Peyote Effect: From the Inquisition to the War on Drugs* (Berkeley: University of California Press, 2018); Chris S. Duvall, *The African Roots of Marijuana* (Durham, NC: Duke University Press, 2019); Haggai Ram, *Intoxicating Zion: A Social History of Hashish in Mandatory Palestine and Israel* (Stanford, CA: Stanford University Press, 2020); James Mills and Patricia Barton, eds., *Drugs and Empires: Essays in Modern Imperialism and Intoxication, c. 1500-c. 1930* (Basingstoke: Palgrave Macmillan, 2007); Thembisa Waetjen, “The Rise and Fall of the Opium Trade in the Transvaal, 1904-1910,” *Journal of Southern African Studies* 43,no.4 (2017): 733-51; Thembisa Waetjen, “Poppies and Gold: Opium and Law-Making on the Witwatersrand, 1904-10,” *Journal of African History* 57, no. 3 (2016): 391-416; Utathya Chattopadhyaya, “Dagga and Prohibition: Markets, Animals, and the Imperial Contexts of Knowledge, 1893-1925,” *South African Historical Journal* 71, no. 4 (2019): 587-613.

11 Martin Holbraad and Morten Axel Pedersen, *The Ontological Turn: An Anthropological Exposition* (Cambridge: Cambridge University Press, 2017); Eduardo Viveiros de Castro, *Cannibal Metaphysics* (Mineeapolis: Univesrity of Minnesota Press, 2015); Marisol de la Cadena, Helene Risør, and Joseph Feldman, “Aperturas Onto-epistémicas: Conversaciones con Marisol delaCadena,” *Antípoda. Revista de Antropologíay Arqueología*, no. 32 (2018): 159-77.

12 Cameron Duff, “Assemblages, Territories, Contexts,” *International Journal of Drug Policy* 33 (2016): 15-20.

13 Here, epistemology and ontology are distinct but contiguous approaches in rewriting the world by asking, “How can the world be different (from the way we know it)?” rather than, “How to know the world?” and “What is the world?” See the work of Marsol de la Cadena.

14 See n. 10.

15 Decolonization, in its own way, has become an omnipresent reference embraced by individuals, groups, and institutions, some of which do not see decolonization as being incompatible with regressive and status quo politics.

16 Achille Mbembe, “Decolonizing Knowledge and the Question of the Archive,” lecture (2015), https://wiser.wits.ac.za/system/files/Achille%20Mbembe%20-%20Decolonizing%20Knowledge%20and%20the%20Question%20of%20the%20Archive.pdf. My contribution does not attempt to speak about the Islamicate world in toto but focuses on the broad and long-lasting influence of Persianate culture and literature in this geocultural space. The sources mobilized in the article are equally varied, and they range from literary and poetical references in Persian and Arabic literatures, medical and scientific treaties, petitions, and historical documents from the thirteenth to the early twentieth centuries. All translations from Arabic, Persian, Italian, Spanish, and French are the author’s work, unless stated otherwise.

17 Siraj Ahmed, *Archaeology of Babel: The Colonial Foundations of the Humanities* (Stanford, CA: Stanford University Press, 2020), 4, 11.

18 Ahmed, *Archaeology of Babel,* 5.

19 Massad, *Islam in Liberalism*, 4.

20 Giorgio Agamben, *Ontologia e politica*, edited by Valeria Bonacci (Rome: Quodlibet, 2019), 130.

21 Agamben, *Ontologia e politica*., 128.

22 de Sousa Santos, *Epistemologies*, 34.

23 Interestingly, Derrida maintained the Orientalist dogma on Islam, saying, “Islam . . . would represent the only religious culture that would have resisted upon until now a European (that is a Greco-Christian and globalatinizing) process of secularization, and thus of democratization, and thus, in strict sense, of politicization”; Massad, *Islam in Liberalism*, 29.

24 Toby Seddon, “Inventing Drugs: A Genealogy of a Regulatory Concept,” *Journal of Law and Society* 43, no. 3 (2016): 393-415.

25 Google NGram Viewer, searching for the term “drug.” https://books.google.com/ngrams/graph?content=drug&year_start=1800&year_end=2019&corpus=26&smoothing=3&direct_url=t1%3B%2Cdrug%3B%2Cc0.

26 Seddon, “Inventing Drugs.

27 In Turkish, the word is *uyuşturucu*, in Urdu *monashiyat*, and in Indonesian *narkoba*.

28 For *taryak* (i.e., “antidote” or “opium” [poison]) as *pharmakon* (scapegoat), see Maziyar Ghiabi, “Ontological Journeys: The Lifeworld of Opium across the Afghan-Iranian Border in/out of the Pharmacy,” *International Journal of Drug Policy* 89 (2021), article 103116, https://doi.org/10.1016/j.drugpo.2021.103116.

29 Franz Rosenthal, “The Herb: Hashish versus Medieval Muslim Society,” in *Man versus Society in Medieval Islam*, edited by Dimitri Gutas (Leiden: Brill, 2015), 153-55.

30 de Sousa Santos, *Epistemologies*, 92-93.

31 See de Sousa Santos, *Epistemologies*, 152-53.

32 Scholarship on alcohol is forgetful of the centrality of alcohol in the Islamic world; global studies of alcohol do not discuss the so-called Middle East or Islamic world, or do so only in passing, as highlighted by Rudi Matthee, *Angels Tapping at the Wine-Shop’s Door: A History of Alcohol in the Islamic Middle East* (London: Hurst, 2023).

33 Mohammed Maraqten, cited in Matthee, *Angels Tapping*, 395.

34 Mahmood Mamdani, *Define and Rule* (Cambridge, MA: Harvard University Press, 2012), 50

35 See Duvall, *African Roots*, 24.

36 Duvall, *African Roots*, 97.

37 See Nina Salouâ Studer’s contribution in this special issue, “‘The Native Is Indeed a Born Addict, but So Far He Has Not Yet Found His True Poison’: Psychiatric Theories on Overconsumption and Race in the Colonial Maghreb,” in this issue.

38 This assertion in itself would be a worthwhile study of drug history but does not fit the purpose of this article.

39 David A. Guba, “Antoine Isaac Silvestre de Sacy and the Myth of the Hachichins: Orientalizing Hashish in Nineteenth-Century France,” *Social History of Alcohol and Drugs* 30, no. 1 (2016): 50-74.

40 For consideration of the power of mythologies in West-East relations, see Billie Jeanne Brownlee and Maziyar Ghiabi, “The Mythological Machine in the Great Civil War (2001-2021): *Oikos* and *Polis* in Nation-Making,” *Middle East Critique* 30, no. 2 (2021): 127-48.

41 Duvall, *African Roots*, 17.

42 Stephanie Honchell, cited in Matthee, *Angels Tapping*.

43 Rudi Matthee, “Alcohol in the Islamic Middle East: Ambivalence and Ambiguity,” *Past and Present* 222, suppl. 9 (2014): 100-25; Rudi Matthee, *The Pursuit of Pleasure: Drugs and Stimulants in Iranian History, 1500-1900* (Princeton, NJ: Princeton University Press, 2009); Rosenthal, “The Herb.”

44 ICR, Dakkha, 6: Laws of Natal 1879-1889, sec. 70, vol. III (Pietermaritzburg: Watson Government Printer). Courtesy of Thembisa Waetjen.

45 See James H. Mills, “Colonial Africa and the International Politics of Cannabis: Egypt, South Africa and the Origins of Global Control,” in Mills and Barton, *Drugs and Empires*, 165-84.

46 Paraphrasing Lüdtke’s suggestions for everyday history; see Alf Lüdtke, *Everyday Life in Mass Dictatorship: Collusion and Evasion* (London: Springer, 2016), 6.

47 William Templer and Alf Ludtke, *The History of Everyday Life: Reconstructing Historical Experiences and Ways of Life* (Princeton, NJ: Princeton University Press, 2018), 7. See Maziyar Ghiabi, ed., “Special Issue: The Everyday Lives of Drugs,” *Third World Quarterly*, forthcoming; Maziyar Ghiabi, “Critique of Everyday Narco-Capitalism,” *Third World Quarterly* (2022), https://doi.org/10.1080/01436597.2022.2053776.

48 Shirazi reported that he discovered the benefits and harms of opium after resorting to eating it on a cold and snowy night that he had to spend outdoors because he had fallen out of the grace with the local governor. He therefore decided to write a medical treaty reflecting on the many dimensions of being an opium “addict.” The treaty is divided into chapters in which the author discusses “Compounds,” “Properties,” “Effects,” “Pleasures,” “Side Effects,” “Benefit,” and other issues including ways to cope with the opium habit. See ‘Emad al-Din Mahmud Bin Mas’ud Shirazi, *Risaleh*-*ye Afyuniyyeh [The Opium Treaty]*, edited by Sadeqpour Omid (1591 (?); Tehran: Alma’i, 2009).

49 ‘Emad al-Din Mahmud Bin Mas’ud Shirazi, cited in Ranin Kazemi, “Doctoring the Body and Exciting the Soul: Drugs and Consumer Culture in Medieval and Early Modern Iran,” *Modern Asian Studies* 54, no. 2 (2020): 582.

50 Seddon, *History of Drugs*.

51 Ralph S. Hattox describes how the month of Ramadan was a busier moment even for coffeehouses in sixteenth-century Cairo, a fact that caused incidents and confrontations, given coffee’s early contentious place in Islamicate social life; Hattox, *Coffee and Coffee-houses: The Origins of a Social Beverage in the Medieval Near East* (Seattle: University of Washington Press, 2014), 84-85.

52 Jahan-’al Azarkhosh, *Afat-e Zendegi [Plague of Life]* (1334; Tehran: Chapp-e Gohar, 1956), 367-68.

53 Azarkhosh, *Afat-e Zendegi*, 373.

54 Azarkhosh, *Afat-e Zendegi*, 510.

55 Hasan Kuhi-Kermani, *Tarikh-e tariyak va taryaki dar Iran* [History of Opium and the Opium Addict in Iran] (1324; Tehran: Chapkhaneh-ye ‘Elmi, 1945).

56 Maziyar Ghiabi, “Drugs and Revolution in Iran: Islamic Devotion, Revolutionary Zeal and Republican Means,” *Iranian Studies* 48, no.2 (2015): 139-63; Maziyar Ghiabi, *Drugs Politics: Managing Disorder in the Islamic Republic of Iran* (Cambridge: Cambridge University Press, 2019).

57 Dawson, *Peyote Effect*, 47.

58 See Ibn Taymiyya, *Le Haschich et l’Extase* (Beyrouth: Albouraq, 2001).

59 Cf. Hattox, *Coffee and* Coffeehouses; Matthee, *Pursuit of Pleasure*.

60 Petition to the Iranian Ministry of Finance from the “70,000 Addicts of Abadan [*mo’tadan-e Abadan*],” 10 October 1941, Asnad-e Arshiv-e Melli (National Archive of Iran), 13619, 22/6/1322.

61 Petition to the Iranian Ministry of Finance, 10 October 1941.

62 It is worth remembering that the Anglo-Persian Oil Company, then Anglo-Iranian Oil Company, became British Petroleum in 1954 (and the National Iranian Oil Company in 1953, followingnationalization). For an overview of Abadan as an oil city, see Rasmus Christian Elling, “Abadan: Oil City Dreams and the Nostalgia for Past Futures in Iran,” Ajam Media Collective, 16 February 2015, https://ajammc.com/2015/02/16/abadan-oil-city-dreams/.

63 Arthur Millspaugh, Response to Petition to the Iranian Ministry of Finance from the “70,000 addicts of Abadan [*mo’tadan-e Abadan*],” 10 October 1941, Asnad-e Arshiv-e Melli (National Archive of Iran), 21480,17/7/1322.

64 This overlap of colonial knowledge, ignorance, and power, and capitalist ethos had parallels across the Global South during the twentieth century, as described by Waetjen in the South African miners’ use of dagga. See Waetjen, “Rise and Fall” and “Poppies and Gold”; Duvall, *African Roots*, 165-66.

65 Maziyar Ghiabi, “The Opium of the State: Local and Global Drug Prohibition in Iran, 1941-1979,” in *The Age of Aryahmer. Late Pahlavi Iran and Its Global Entanglements*, edited by Roham Alvandi (London: Gingko Library, 2018), 70-109.

66 Previous bans on opium and other substances were temporary and mostly concerned with moral punishment of public intoxication as opposed to a systematic decision to pro-hibit the use of opiates or to eradicate the poppy plant. See Matthee, *Pursuit of Pleasure*.

67 *New York Times*, 11 February 1973, cited in Ghiabi, “Opium of the State.”

68 See Alex Mold, “The ‘British System’of Heroin Addiction Treatment and the Opening of Drug Dependence Units, 1965-1970,” *Social History of Medicine* 17, no. 3 (2004): 501-17.

69 International Narcotics Control Board, “Report of the International Narcotics Control Board for 2001 “ (2002), https://www.incb.org/incb/en/publications/annual-reports/annual-report-2001.html.

70 Central Treaty Organisation, “Seminar on Public Health and Medical Problems Involved in Narcotics Drug Addiction” (8-12 April 1972, Tehran), https://pdf.usaid.gov/pdf_docs/PNABI608.pdf; emphasis added.

71 Isaac Campos, “Mexicans and the Origins of Marijuana Prohibition in the United States: A Reassessment,” *Social History of Alcohol and Drugs* 32, no. 1 (2018): 6-37; Campos, “ADip-lomatic Failure: The Mexican Role in the Demise of the 1940 Reglamento Federal de Toxicomanías,” *Third World Quarterly* 39, no. 2 (2018): 232-47.

72 Intersecting figures are also the *qalandar* and the *malamati*. I will refer to *rend* as the catchall term for all three figures. Often described as “rake, ruffian, pious rogue, brigand, libertine, lout, debauchee,” the *rend* is an “antithesis of establishment propriety.” The variations among these terms can be substantial only when considering the specific polemics between mystical orders such as *qalandar* and *malamati*. Whereas the *qalandar* and *malamati* were actual spiritual orders, the *rend* refers to an ethics of life unconforming to orthodox religiosity and social conventions—thus my preference for this term over the others in the text. All three terms have strong historical association to “intoxication” and pursuit of pleasure. See Ehsan Yarshater, Baha’-al-Din Khorramshahi, J. T. P. de Bruijn, Daniela Meneghini Correale, Julie Scott Meisami, Franklin Lewis, Parvin Loloi, et al., “Hafez,” in *Encyclopaedia Iranica Online* (Leiden: Brill, 2020), https://doi.org/10.1163/2330-4804_EIRO_COM_2596. For an overview, see Leonard Lewisohn, *Hafiz and the Religion of Love in Classical Persian Poetry* (London: Bloomsbury, 2010).

73 Ahmed, *Archaeology of Babel*, 89.

74 Hafez himself is a nom de plume (*takhallos*) that means “Koran-reciter,” making the heterodoxy of the poet’s verses all the more evident.

75 Ahmed, *Archaeology of Babel*, 89.

76 It is the least Western of contemporary philosophers who has engaged with a form of life in a distant dialogue with what I elaborate here; see Giorgio Agamben, *Altissima povertà: regolemonastiche e forma di vita; Homo sacer, IV, I* (Milan: Pozza, 2011). This is also somehow reflected in the ontological turn within anthropology, feminist theory, and science and technology studies (STS), which have, in different ways, been reclaiming the legitimacy of non-Western thought, less through actual paradigmatic exposition than by means of critique.

77 For a detailed analysis of the metaphorical and historical figures outlined in this section, refer to Encyclopaedia Iranica, “Hafez viii. Hafez and Rendi,” https://iranicaonline.org/articles/hafez-viii.

78 For an excellent article on premodern Iranian use of drugs, see Kazemi, “Doctoring the Body.”

79 Hafez, ghazal no. 44.

80 Interestingly some of these boundaries have been contested by medical anthropological research on “drugs” in ways that illuminate this discussion, even though they differ contextually. See the authors in the edited volume by Eugene Raikhel and William Garriott, *Addiction Trajectories* (Durham, NC: Duke University Press, 2013). It is telling that this excellent volume does not include any chapter on the Islamicate world. So far, this has been the rule in the broad field of drug studies; cf. Suzanne Fraser, David Moore, and Helen Keane, *Habits: Remaking Addiction* (London: Palgrave Macmillan, 2014); Mariana Valverde, *Diseases of the Will: Alcohol and the Dilemmas of Freedom* (Cambridge: Cambridge University Press, 1998); Nancy D. Campbell, *Using Women: Gender, Drug Policy, and Social Justice* (New York: Routledge, 2002); Nancy D. Campbell, *Discovering Addiction: The Science and Politics of Substance Abuse Research* (Ann Arbor: University of Michigan Press, 2007).

81 Hafez, ghazal no. 393.

82 Hafez, ghazal no. 245.

83 The courts and the elites have historically indulged in wine drinking and consumption of mind-altering substances. For a detailed account of high-society drug taking, see Matthee, *Pursuit of Pleasure*.

84 This concept was further theorized and popularized by Escobar, *Pluriversal Politics*.

85 For an overview of the Islamic concept of *dhawq*, see Stephen Hirtenstein, “Dhawq,” in *Encyclopaedia Islamica* (2018), https://doi.org/10.1163/1875-9831_isla_COM_037167.

86 Escobar, *Pluriversal Politics*; de Sousa Santos, *Epistemologies*; Mignolo and Walsh, *On Decoloniality*.

87 Kazemi, “Doctoring the Body,” 588.

88 Obeyd-e Zakani, ghazal no. 49.

89 Hafez, ghazal no. 393.

90 Parvin E’tesami, “The Drunk and the Sober,” https://ganjoor.net/parvin/divanp/mtm/sh142%20/.

91 I am grateful to the reflections provided by Rashid Kakavand during an interview on the Iranian TV program “Ketabbaz.”

92 See Mbembe, “Decolonizing Knowledge,” 6.

93 Frantz Fanon, *The Wretched of the Earth*, translated by Richard Philcox (1961;NewYork: Grove, 2004), 6.

94 In this regard, the emerging fields of medical anthropology of drugs and addiction and STS-informed drug theory has great potential for informing history writing on intoxication.

95 Lucas Richert, *Strange Trips: Science, Culture, and the Regulation of Drugs*, vol. 51 (Montreal: McGill-Queen’s University Press, 2018); David Herzberg, *White Market Drugs: Big Pharma and the Hidden History of Addiction in America* (Chilcago: University of Chicago Press, 2020).

96 Escobar, *Pluriversal Politics*, 21,41.

97 Ghiabi, “Critique.”

98 Escobar, *Pluriversal Politics*, 74.

